# Entorhinal cortex–hippocampal circuit connectivity in health and disease

**DOI:** 10.3389/fnhum.2024.1448791

**Published:** 2024-09-20

**Authors:** Melissa Hernández-Frausto, Carmen Vivar

**Affiliations:** ^1^NYU Neuroscience Institute, Department of Neuroscience and Physiology, NYU Grossman School of Medicine, New York University Langone Medical Center, New York, NY, United States; ^2^Laboratory of Neurogenesis and Neuroplasticity, Department of Physiology, Biophysics and Neuroscience, Centro de Investigacion y de Estudios Avanzados del Instituto Politécnico Nacional, Mexico City, Mexico

**Keywords:** entorhinal cortex, hippocampus, neuromodulation, episodic memory, Alzheimer’s disease, exercise, aging

## Abstract

The entorhinal cortex (EC) and hippocampal (HC) connectivity is the main source of episodic memory formation and consolidation. The entorhinal–hippocampal (EC-HC) connection is classified as canonically glutamatergic and, more recently, has been characterized as a non-canonical GABAergic connection. Recent evidence shows that both EC and HC receive inputs from dopaminergic, cholinergic, and noradrenergic projections that modulate the mnemonic processes linked to the encoding and consolidation of memories. In the present review, we address the latest findings on the EC-HC connectivity and the role of neuromodulations during the mnemonic mechanisms of encoding and consolidation of memories and highlight the value of the cross-species approach to unravel the underlying cellular mechanisms known. Furthermore, we discuss how EC-HC connectivity early neurodegeneration may contribute to the dysfunction of episodic memories observed in aging and Alzheimer’s disease (AD). Finally, we described how exercise may be a fundamental tool to prevent or decrease neurodegeneration.

## 1 Introduction

Formation and consolidation of new memories are critical brain functions that arise on the limbic system (MacDonald et al., 2011; Manns and Eichenbaum, 2006) and require the association of salient spatial, temporal, and contextual sensory elements within an environment (Basu et al., 2016; Fortin et al., 2004). Those properties rely on the appropriate function and connectivity between the entorhinal cortex (EC) and the hippocampal area (HC), as a reciprocal entorhinal cortex–hippocampal loop where EC sends and receives projections from HC (Basu and Siegelbaum, 2015; Witter et al., 2017). The interaction between EC and HC promotes a sequential organization that leads to the formation and consolidation of episodic memories of people, places, objects, events, and locations in space (Basu et al., 2016; Basu and Siegelbaum, 2015; Brun et al., 2008; Buzsáki and Moser, 2013; Igarashi et al., 2014; Ye et al., 2018). An understanding of this connectivity at molecular, cellular, circuitry, and functional levels could provide insights into the formation and consolidation of new memories. Additionally, given that the EC is one of the first brain regions to exhibit neurodegeneration in Alzheimer’s disease (Igarashi, 2023), identifying the properties of the connectivity between EC and HC could also provide insights into disease mechanisms.

In this review, we focus on the role of neuromodulators in entorhinal cortex–hippocampal functional connectivity during the encoding and consolidation of memories. We highlight the value of animal research to unravel the underlying cellular mechanisms of encoding and consolidation of new memories. We discuss how neurodegeneration of the entorhinal cortex–hippocampal connectivity during aging may contribute to Alzheimer’s disease dysfunction and how exercise may prevent or decrease this neurodegeneration.

## 2 Entorhinal cortex–hippocampal architecture

The EC serves as the main input and output of the HC area and works as the nodal point for other cortical areas (Basu and Siegelbaum, 2015; Canto et al., 2008; Witter et al., 2017). The EC is located in the medial temporal lobe and is surrounded by the olfactory cortex, amygdala, parasubiculum, and perirhinal cortex (Canto et al., 2008). Neuroanatomical evidence from studies in rodents suggests that EC is subdivided into two major subregions, the medial- (MEC) and lateral entorhinal cortex (LEC) (Canto et al., 2008; Witter et al., 2017), which convey mainly spatial and non-spatial features, respectively, into the HC (Knierim et al., 2013; Witter et al., 2017). For many years, researchers assumed that the connectivity of the human EC was similar to that observed in rodents. It wasn’t until recently that the use of ultra-high-resolution fMRI confirmed the functional division of human EC into two subregions, the anterior–lateral and posterior–medial regions with object-related and spatial preferences, respectively, in line with the animal literature (Maass et al., 2015; Navarro Schröder et al., 2015; Schultz et al., 2015). Thus, the functional connectivity of the human EC closely parallels the anatomical connectivity patterns of the rodent and non-human primate EC.

Anatomically, MEC is highly innervated by the presubiculum and postrhinal cortex (in rodents; parahippocampal cortex in primates) and processes spatial information. The MEC cells exhibit spatial and directional firing patterns, such as the widely studied grid cells, heading direction cells, speed cells, and border cells (Fyhn et al., 2004; Hafting et al., 2005; Kropff et al., 2015; Moser et al., 2008; Sasaki et al., 2015; Solstad et al., 2008). By contrast, LEC is vastly innervated by the olfactory bulb and perirhinal cortex. The LEC neurons respond to non-spatial features of the environment such as odors, objects, and timing (Basu and Siegelbaum, 2015; Deshmukh and Knierim, 2011; Wan et al., 1999; Sasaki et al., 2015; Tsao et al., 2013, 2018; Zhu et al., 1995). While MEC is traditionally thought to be highly innervated by the presubiculum and postrhinal cortex and LEC from the olfactory bulb and perirhinal cortex, this prevailing concept has been recently challenged using tract tracing. This new technique revealed that the postrhinal cortex preferably targets the LEC instead of the MEC, thus converging postrhinal and perirhinal projections on LEC layer II (Doan et al., 2019).

This complex anatomical connectivity suggests that MEC and LEC cannot be understood simply as a spatial-non-spatial dichotomy, but rather as a potential site of integration for information about a multitude of different environmental features relevant to spatial location. Indeed, it has been proposed that MEC may be involved in path integration based on a global frame of references using internal self-motion cues and external inputs about the environment. Thus, MEC may provide the hippocampus with information on the spatial context of an experience. The LEC may process information about individual item features and locations based on a local landmark of reference using external sensory inputs, providing the hippocampus with information about the content of an experience (Knierim et al., 2013; Keene et al., 2016). Whether this complex anatomical and functional organization of EC takes place in humans is still largely unknown. However, a thorough anatomical and functional characterization of EC subfields may not only advance our understanding of human memory processing but also have important clinical implications in neurodegenerative diseases.

Within laminar classification, both the LEC and the MEC are mainly sectioned into five well-defined layers (layers I, II, III, IV, and V), with an extra layer VI that has not been completely studied. Nevertheless, this distribution is consistent and remains among different species, including rodents, non-human primates, and humans (Braak and Braak, 1992; Ohara et al., 2018, 2021; Witter et al., 2017). Additionally, connectivity with HC is widely different across layers.

The most superficial layers II and III are thought to provide the main input to the HC, whereas deeper layers V and VI receive the outputs from HC and subiculum (Ohara et al., 2018, 2021; Roy et al., 2017; Witter et al., 2017). Functional studies in humans show that during the performance of a memory task, the superficial layers of the EC have significantly greater activation associated with task-related encoding conditions, whereas the deep layers show significantly greater activation associated with task-related retrieval conditions (Maass et al., 2014; Zhang et al., 2023). Future functional studies examining alterations in task-related encoding and retrieval will be key in understanding the early pathology accumulation in the EC layers during the progress of AD-related memory impairment.

The use of molecular markers, electrophysiological, and anatomical tools has allowed the identification of neurons with layer-specific features and distinct molecular phenotypes. In rodents, in layer II of MEC, there is a robust presence of pyramidal neurons and large multipolar neurons denominated stellate cells (SCs), while in LEC, there is a presence of pyramidal neurons, medium-sized multipolar cells, and large-sized denominated fan cells (FCs) (Witter et al., 2017). Neurochemically, there are two types of neurons in layer II of both subregions of EC: calbindin-and reelin-expressing cells that are distinctively organized. Calbindin is a member of the calcium-binding proteins and has a critical role in preventing neuronal death as well as maintaining calcium homeostasis, whereas reelin is a glycoprotein implicated in synaptic plasticity (Schmidt, 2012; Stranahan et al., 2013). In rodents’ MEC, calbindin^+^ and reelin^+^ cells appear to be grouped in patches, whereas in LEC the two cell types are confined to two separate sublayers, reelin cells in layer IIa and calbindin cells in layer IIb. Reelin^+^ neurons located in both MEC and LEC project to the dentate gyrus (DG) and CA3 area, whereas calbindin^+^ neurons project to the area CA1, the contralateral EC, olfactory bulb, and the piriform cortex (Fuchs et al., 2016; Kitamura et al., 2014; Ray et al., 2014; Witter et al., 2017).

On the other hand, the HC is located in the temporal lobe and is a multilayered structure composed primarily of densely packed neurons. Similar to EC, the HC is subdivided into regions classified as the DG, the proper HC that consists of *Cornu Ammonis* areas 1–3 (CA1, CA2, and CA3), and the subiculum (sub) (Amaral and Witter, 1989). In humans, the HC is a widely elongated structure, whereas in rodents, it is large but less elongated with a cashew-similar form (Knierim, 2015; Zemla and Basu, 2017). Nevertheless, it is believed their function and basic structures are maintained among mammals, including rodents and humans.

The DG is thought to contribute to the formation of new episodic memories, novelty, and pattern separation (Bakker et al., 2008; Goodrich-Hunsaker et al., 2008; Rolls, 2016; Leutgeb et al., 2007). Anatomically, the DG consists of three distinct layers, an outer molecular layer, a middle granule cell layer, and an inner polymorphic layer or hilus. The principal cells of the DG are the granule cells that, packed together, give shape to the granule cell layer. The molecular layer is subdivided into three segments, the middle and outer molecular layers that receive the afferent projections from MEC and LEC, respectively, and the inner molecular layer that receives inputs primarily from the GABAergic interneurons, mossy cells, and subcortical modulatory inputs such as the medial septum and diagonal band of Broca (Amaral et al., 2007). The axons of the granule cells, known as mossy fibers, project to the dendrites of CA3 pyramidal neurons.

The DG is one of the few brain areas that continuously generate new granule cells throughout life in mammalian species, a physiological phenomenon denominated “adult neurogenesis” which is highly reproducible in rodent models (Ming and Song, 2005; Vivar and van Praag, 2013), whereas in humans, it is still under debate (Sorrells et al., 2018; Snyder, 2019; Moreno-Jiménez et al., 2021). The neural stem cells that give rise to the new granule cells are located in the subgranular zone. These new granule cells are integrated into the hippocampal network and exhibit enhanced excitability and plasticity compared to developmentally generated granule cells (Schmidt-Hieber et al., 2004; Ge et al., 2007; Ming and Song, 2005). Continuous addition of easily excitable new granule cells to the DG suggests a unique contribution to memory function, with neurogenesis as a mechanism underlying efficient cortical storage of new memories and pattern separation (Kitamura et al., 2009; Clelland et al., 2009; Creer et al., 2010; Sahay et al., 2011; Nakashiba et al., 2012; Kempermann, 2022).

**Figure 1:**
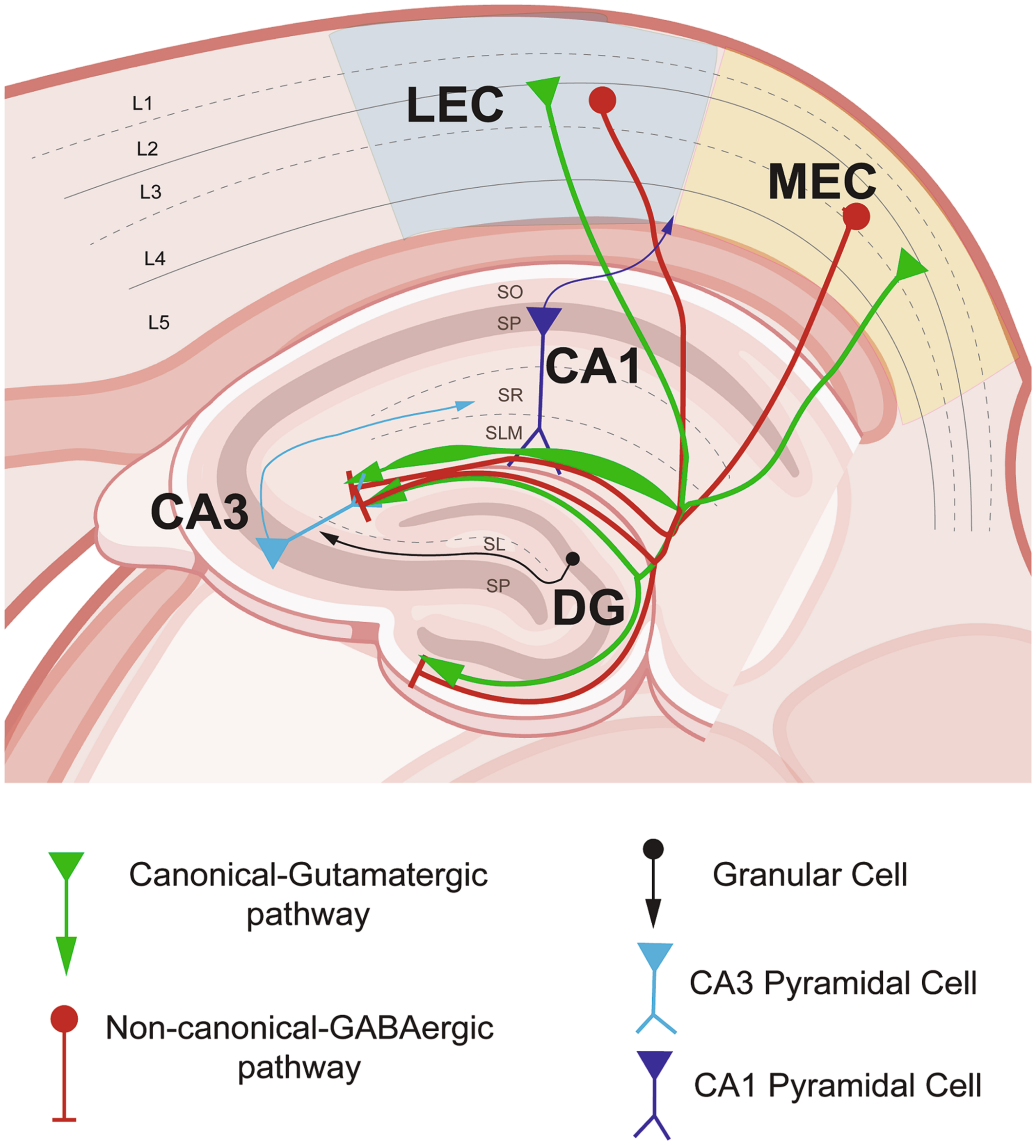
Entorhinal cortex–hippocampal circuit through the canonical-glutamatergic and non-canonical GABaergic pathways in non-pathological conditions. Schematic representation of the entorhinal cortex inputs from lateral- (LEC) and medial entorhinal cortex (MEC) into the hippocampal area. In green, the canonical–glutamatergic pathway, showing the variability of the density of projections in the stratum lacunosum moleculare (SLM) layer of the hippocampus (HC). In red, the non-canonical–GABAergic pathway originated from LEC and MEC running into the HC area, please note the differential distribution among the SLM layer. Figure partially created with BioRender and based on Hernández-Frausto et al., 2023.

In the HC, the layers of the CA areas are divided according to the localization of cell bodies, axons, and dendrite localizations. The somata of the pyramidal cells constitute the *stratum pyramidale* (SP). The *stratum oriens* (SO) contains the basal dendrites of the pyramidal cells, whereas the *stratum lucidum* (SL) is composed of the axons from DG granular cells, which project straight to CA3 basal dendrites. The *stratum radiatum* (SR) is confirmed by the apical dendrites of the pyramidal cells and is the area where axons from CA3 pyramidal cells project to CA1 via the Schaffer collaterals (SC). Finally, the stratum lacunosum moleculare (SLM) is the layer where the most distal dendrites are located and where the EC of the MEC and LEC inputs are reached (Amaral and Witter, 1989; Basu and Siegelbaum, 2015; Witter et al., 2000) (Figure 1).

In addition to the classical role of the principal excitatory neurons to shape episodic memory formation, recent evidence has increased attention to the function of GABAergic interneurons (GABAergic INs) in modulating the EC-HC circuitry function. In physiological conditions, they work to maintain the excitation–inhibition balance by shaping, acquiring, and transforming the information of the glutamatergic neurons through synaptic transmission and plasticity (McBain and Fisahn, 2001). In the cortex, GABAergic INs constitute around 10–20% of all neurons, whereas in the hippocampus, they are around 10–15% (Bezaire and Soltesz, 2013; Pelkey et al., 2017). They are classified according to their morphology, molecular markers and neurochemical content, intrinsic properties, developmental origin, and function (Petilla Interneuron Nomenclature Group, 2008; Harris et al., 2018; Klausberger and Somogyi, 2008; Le Magueresse and Monyer, 2013; Pelkey et al., 2017). A recent classification is in terms of their neurochemical content, with the presence of calcium-binding proteins and molecular markers such as parvalbumin (PV), calbindin (CB) and calretinin (CR), cholecystokinin (CCK), somatostatin (SST), vasoactive intestinal peptide (VIP), neuropeptide Y (NPY), and neuron-derived neurotrophic factor (NDNF) (Hernández-Frausto et al., 2023; Klausberger and Somogyi, 2008; McBain and Fisahn, 2001). Thus, the cytoarchitecture of the EC-HC circuit is orchestrated not only for the influence of principal cells but also for the important role of GABAergic INs in maintaining the excitation–inhibition balance of the circuit.

## 3 Information flow in the entorhinal cortex–hippocampal circuit

### 3.1 Canonical pathways—glutamatergic inputs

The processing of multimodal sensory information in the EC and HC is required for episodic memory formation. The general canonical model of the EC-HC circuit posits that HC receives multisensory inputs from EC layers II and III directly to the CA1 area and DG. The first and most studied is the trisynaptic pathway (EC layer II → DG → CA3 → CA1), where the granule cells receive direct excitatory projections from MEC and LEC layer II. However, transynaptic tracing studies show that particularly adult-born granule cells receive direct innervation preferentially from the LEC layer II and the perirhinal cortex rather than from the MEC (Vivar et al., 2013). It suggests that new granule cells may be more specialized in the processing of incoming environmental information than developmentally generated granule cells that are innervated similarly by both LEC and MEC. Indeed, disruption of adult-born granule cell circuitry by LEC-perirhinal cortex lesion led to deficits in fine discrimination (Vivar et al., 2013).

Next, the dentate granule cells route the processed information via mossy fibers to the CA3 area, connecting the proximal portion of pyramidal cells´ dendrites. The CA3 pyramidal neurons then project their axons called “Schaffer collateral” to the medial portion of CA1 pyramidal cells´ dendrites. Finally, CA1 pyramidal cells and subiculum neurons send back processed information to layer V of EC and other brain structures (van Strien et al., 2009; Basu and Siegelbaum, 2015; Witter et al., 2000).

The HC uses sparse population activity to process information in memory, recruiting only a small proportion of neurons simultaneously (Buzsáki and Moser, 2013). This process is supported by DG, which sends sensory information to the HC, decorrelating these inputs into non-overlapping patterns and allowing sparse hippocampal activation (Treves and Rolls, 1994; Knierim and Neunuebel, 2016). Thus, DG-CA3 connectivity allows the distinction of new memories from older ones and similarly enables contextual and spatial representations, pattern separation, and the encoding and retrieval of episodic memories (Knierim and Neunuebel, 2016). Meanwhile, CA3 recurrent synaptic connections appear necessary for pattern completion and memory recall (Guzman et al., 2016). Recent evidence shows that dentate adult-born granule cells support sparser hippocampal population activity. By combining triple ensemble recordings in DG, CA3, and CA1, McHugh et al. showed that adult-born granule cells (4–7 weeks old), with enhanced activity responses to novelty and strong modulation by theta oscillations, increase the sparsity of hippocampal population patterns. Contrarily, adult-born neuron suppression reduces the hippocampal sparsity, inducing the increment of principal cell firing rate in DG, CA3, and CA1 and impairing novel object recognition (McHugh et al., 2022). Thus, effective mnemonic processing of new information may require high-firing neurons with preferential input from LEC to promote sparse hippocampal population activity.

The second pathway is the direct or disynaptic pathway to CA1 (EC layer III → CA1). In this pathway, glutamatergic neurons from LEC and MEC layer III send their axonal projections directly to the distal portion of CA1 pyramidal cells (Amaral and Witter, 1989; Deshmukh and Knierim, 2011; Ohara et al., 2018; Suh et al., 2011). These inputs are not homologous among the LEC and MEC. Anatomical tracing studies together with *in vitro* electrophysiology, two-photon microscopy spine imaging, and optogenetics in rodents have shown that direct inputs from MEC and LEC are distinct in CA1 through the transverse axis, with densest MEC projections in proximal CA1 and LEC denser to distal CA1 (Masurkar et al., 2017). Moreover, the direct MEC inputs to CA1 preferentially excite deep pyramidal neurons in the proximal axis, whereas direct LEC inputs preferentially excite superficial pyramidal neurons more distally located (Masurkar et al., 2017). This differential connectivity may lead to a mechanism where spatial and non-spatial memory are preferentially targeted (Figure 1).

### 3.2 Non-canonical pathways—GABAergic inputs

Recent evidence suggests that GABAergic neurons in the EC not only act locally to maintain the excitation–inhibition balance through inhibition or disinhibition of microcircuits as we described above. The GABAergic neurons also send long-range GABAergic projections (LRGPs) to synchronize activity across longer distances to coordinate and act typically as a disinhibitory local circuit, as they usually target local GABAergic IN (Basu et al., 2016; Caputi et al., 2013; Hernández-Frausto et al., 2023; Melzer et al., 2012; Melzer and Monyer, 2020). Similar to the canonical-glutamatergic inputs, LRGPs from the LEC and MEC project through two pathways to CA1 either indirectly, crossing first DG → CA3 pyramidal neurons to finalize in CA1 pyramidal neurons, or directly to CA1 pyramidal neurons (EC → CA1) (Basu et al., 2016; Melzer et al., 2012). Although the pathways of projections are well described, the specific location of these GABAergic IN in MEC and LEC layers remains elusive. According to the HC distribution, these non-canonical GABAergic inputs are not homologous through the transverse axis in CA1. While the densest projections from MEC are located mostly in proximal CA1, the LEC projections are equally dense among the proximal–distal CA1 axis (Basu et al., 2016; Melzer et al., 2012). In line with their function, anatomical retrograde labeling studies show that LRGPs from EC project into the HC (Germroth et al., 1989). Specifically, the LRGPs from MEC are mostly located in dorsal and intermediate HC. LRGPs’ axons originate from PV^+^ interneurons and other undetermined GABAergic subtypes that modulate local GABAergic neuron theta synchrony in the HC (Melzer et al., 2012). Furthermore, the LRGPs from LEC are highly connected to CCK^+^ interneurons in CA1. They are highly activated by behaviorally salient cues such as water rewards and air puffs and are important for novelty and contextual salience discrimination (Basu et al., 2016). Thus, the canonical and non-canonical pathways of the entorhinal cortico-hippocampal circuit may contribute differentially to the processing of multisensory information and collectively encode them as long-term memories (Figure 1). Although these pathways have been described in rodents and are relatively conserved across mammals, human studies are still needed to determine the specificity of the canonical-glutamatergic and non-canonical GABAergic pathways (Bergmann et al., 2016).

## 4 Entorhinal cortex–hippocampal circuit neuromodulation in health

While the EC-HC circuit has been studied across its own glutamatergic and GABAergic pathways, both structures are also highly innervated from different cortical and subcortical brain regions. The EC-HC circuit receives massive neuromodulatory fibers from cholinergic, noradrenergic, and dopaminergic inputs highly involved in the functionality of episodic memory formation and recall. Here, we will describe the specificities of the neuromodulatory inputs received and their role in shaping episodic memory formation and recall.

### 4.1 Acetylcholine

Acetylcholine (ACh) plays an important role in learning and memory, having a differential role in the encoding and consolidation of memories, showing high levels of activity during memory encoding and low levels of activity in memory consolidation (Haam et al., 2018; Bartus et al., 1982). Across mammal species, specifically in humans and rodents, the EC receives profuse cholinergic innervation from the medial septum (MS) and the diagonal band of Broca (DBB) that underlies working memory, spatial processing, and episodic memory (Heys et al., 2012; De Lacalle et al., 1994). The ACh exerts differential effects depending on the EC circuit. For example, ACh influences the intrinsic firing pattern of MEC layer V stellate cells, which generates graded persistent activity. This activation mechanism constitutes an elementary mechanism for working memory (Egorov et al., 2002). Whereas in grid cells, the activation of muscarinic receptors modulates their periodic spatial tuning, which may provide a coordinate system for navigation behavior and memory formation (Newman et al., 2014). Similar to EC, the HC is highly innervated by MS and DBB (De Lacalle et al., 1994; Haam and Yakel, 2017). These inputs to HC play a role in generating and pacing theta-band oscillations during novelty detection, exploration, and the formation and consolidation of episodic memory (Haam and Yakel, 2017). In rodents, stimulation of MS cholinergic neurons induces changes in the HC interneuron firing activity, with the concomitantly decreased firing of principal cells (Dannenberg et al., 2015). Moreover, MS cholinergic neurons modulate HC theta-band oscillations *in vivo* (Dannenberg et al., 2015). Within the hippocampal area, computational modeling and experimental studies show that ACh facilitates afferent projections of the perforant path into CA1, acting as the active modulator during encoding mechanisms of external pathways while inhibiting the local intrinsic pathways that are part of memory consolidation, such as the back projections from HC to EC (Hasselmo, 1999; Newman et al., 2012; Haam et al., 2018). Thus, circuits that carry extrinsic information are preferentially activated, while the intrinsic projections are toned down.

Although few studies compare the influence of ACh in the EC-HC circuitry loop, Haam et al. analyzed the cholinergic projections from MS into CA1. They observed a prominent innervation of ACh projections onto OLM interneurons, which in turn decreased the activity of the excitatory output of the HC. Furthermore, it was shown that in deep layers of EC, the inactivation of ACh projections is critical for proper memory formation (Haam et al., 2018). More recently, Palacios-Filardo et al. (2021) tested the hypothesis that the release of ACh into the hippocampus has a stronger connection in the direct EC to CA1 pathway over the local pathway of Schaffer Collateral (SC). They found that the release of ACh decreases SC and EC excitatory inputs similarly, but with higher sensitivity of EC inputs to cholinergic modulation, resulting in an increased excitation–inhibition ratio.

### 4.2 Noradrenaline

Noradrenaline (NA)-containing fibers originated in the locus coeruleus (LC) that innervate wide cortical and subcortical structures. It is known that the LC neurons respond to novel or salient events (Segal and Bloom, 1974; Bacon et al., 2020; Breton-Provencher and Sur, 2019; Breton-Provencher et al., 2021). In the HC, all subregions receive dense noradrenergic innervation mainly from the LC (Walling et al., 2012; Wagatsuma et al., 2018). The inactivation of the LC inputs into the HC induces the impairment in memory and learning, whereas stimulation of LC fibers reorganizes place cell representations and enhances memory in the HC (Compton et al., 1995; Kaufman et al., 2020). However, in the EC, the noradrenergic modulation is less known. Studies in rodents show that the application of NA induces hyperpolarization and decreases the excitability of the EC superficial layers neurons, with no effects on neuronal excitability in the deep layers (Xiao et al., 2009). Stress studies are known to elicit spatial memory deficits, inducing LC NA release on EC to increase the frequency and amplitude of spike-driven inhibitory postsynaptic currents (IPSCs) in MEC layer II cells (Hartner and Schrader, 2018). These effects are primarily mediated by α1 and α2 adrenergic receptors and *β*-adrenergic receptors in EC (Boyajian et al., 1987; Unnerstall et al., 1984). Interestingly, when NA modulation of the entorhinal cortico-hippocampal circuit was evaluated, results showed that NA influences mainly LEC rather than MEC inputs to DG and CA1 (Ito and Schuman, 2012), suggesting that NA may allow differential encoding of non-spatial information in the HC by controlling LEC inputs. Given the impact of noradrenergic modulation in the entorhinal cortico-hippocampal circuit, further studies are needed to understand the modulatory noradrenergic mechanisms underlying the formation and consolidation of new memories.

### 4.3 Dopamine

Memory encoding and consolidation processes have been linked with dopaminergic signaling for a long time. In humans, dopamine (DA) neurons from the ventral tegmental area (VTA) project to the hippocampus (Ruiz-Tejada et al., 2022). However, it is still unclear how DA influences the entorhinal cortico-hippocampal circuit. A recent study in rodents found that dopaminergic inputs from VTA are important for the modulation of memory encoding in LEC (Lee et al., 2021). The specific inhibition of fan cells in LEC impaired the learning of new associations while sparing the retrieval of pre-learned memory. Moreover, the inhibition of LEC DA signals disrupted the associative encoding of fan cells and impaired learning performance (Lee et al., 2021). Thus, the DA regulation in LEC may contribute to representing cognitive maps of abstract task rule domains.

In the HC, especially the dorsal region, DA neurotransmission is critical for episodic memory (Kempadoo et al., 2016; Takeuchi et al., 2016; Wilmot et al., 2024). The VTA is the presumed source of DA in the dorsal HC. However, recent evidence shows that axons from LC neurons release DA in the dorsal HC to enhance selective attention and spatial object recognition via the dopamine D1/D5 receptors (Kempadoo et al., 2016; Chowdhury et al., 2022). Additionally, DA influx from the VTA into the HC is critical for novelty, as a predictor error, and for spatial long-term memory retrieval with the facilitation of LTP (Broussard et al., 2016; Chowdhury et al., 2022; Huang and Kandel, 2006; Broussard et al., 2012; Lisman and Grace, 2005; Navakkode et al., 2010; Velazquez-Delgado et al., 2024). The LC (the noradrenergic principal hub) also sends dopaminergic projections to the dorsal portion of CA1, which has a principal role in contextual memory linking. The DA from LC modulates the excitability of CA1 pyramidal neurons for memory ensembles and the stability of these ensembles, leading to a link in the neuromodulatory system that affects memory linking without memory formation (Chowdhury et al., 2022).

Thus, DA modulation at EC and HC levels may favor the memory encoding and consolidation process. In addition, evidence of the DA-modulatory EC-HC connectivity shows that DA influences mainly LEC inputs to DG and CA1, similar to NA (Ito and Schuman, 2012), supporting the role of LEC and DA modulation on memory encoding (Lee et al., 2021). Therefore, DA may provide a differential regulation of two information streams (spatial and non-spatial) from the EC to the HC.

**Figure 2:**
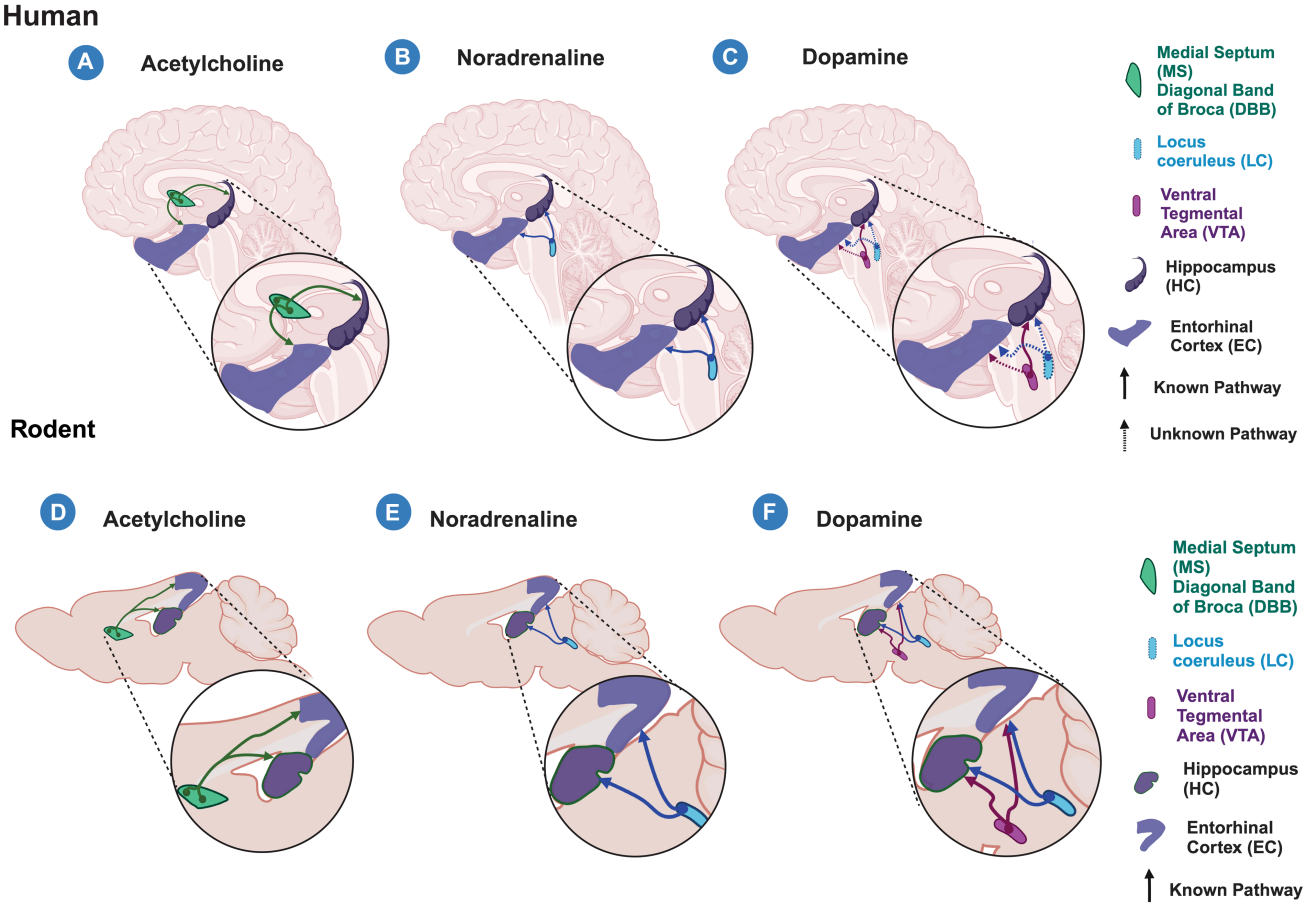
Acetylcholine, Noradrenaline and Dopamine inputs to the entorhinal cortex-hippocampal circuit in humans and rodents. Schematic representation of known (solid lines) and unknown (dashed lines) modulatory inputs to the entorhinal cortex (EC) and hippocampus (HC) in humans **(A–C)** and rodents **(C–F)**. **(A–C)** in humans, acetylcholine released from the medial septum (MS) and diagonal band of Broca (DBB) **(A)** and noradrenaline released from the Locus Ceruleus (LC) **(B)** modulate both the EC and HC areas. **(C)** Dopamine released from the Ventral Tegmental Area (VTA) modulates hippocampal processing, however, it is unknown whether it does so in the EC. In humans, it is unknown whether EC and HC receive dopaminergic modulation from the LC as is observed in rodents. **(D–F)** In rodents, the entorhinal cortex-hippocampal circuit is modulated by the cholinergic input from MS and DBB **(D)**, noradrenergic input from LC **(E)**, and dopaminergic inputs from the VTA and LC **(F)**. Created with BioRender.

Altogether, this suggests that massive ACh, NA, and DA inputs to the entorhinal cortico-hippocampal circuit differentially modulate this circuit, highlighting the selective modulation of LEC projections to DG and CA1. Nevertheless, it is important to note that most of the recent studies are based on rodent models and non-human primate research, and while they allow us to improve our understanding of the formation and consolidation of new memories, they create a starting point for future research in humans. Moreover, this information has been generated by studying the canonical pathway; however, it is still largely unknown how the non-canonical pathway modulation may impact the encoding of new memories. Therefore, studies are needed to understand the role of these inputs in the encoding of new memories in humans and to determine whether the cross-species counterpart analyses are comparable (Figure 2).

## 5 Entorhinal cortex–hippocampal circuit during aging and disease

During aging, there is a significant decline in episodic memory that is observed from middle age and is accelerated by the carriage of the ε4 allele of the apolipoprotein E (APOE) gene (Salthouse, 2019; Beydoun et al., 2021; Dohm-Hansen et al., 2024). Age-associated cognitive impairment has been described in multiple species, including rats, macaque monkeys, and humans (Gallagher and Rapp, 1997). These cognitive alterations are accompanied by structural, cellular, molecular, and functional changes that disrupt the cortico-hippocampal circuitry (Stranahan and Mattson, 2010; Zhao et al., 2022). Anatomical changes in EC and HC have been associated with age-related declines in memory performance. Specifically in the EC, its shrinking has been linked to a memory performance decline in adults who are at increased risk of developing dementia, elderly, and concurrent AD patients (de Toledo-Morrell et al., 2000; Dickerson et al., 2001; Du et al., 2003; Olsen et al., 2017). Moreover, in healthy adults, mild shrinkage of the EC may be a sensitive predictor of memory decline (Rodrigue and Raz, 2004). In the HC, a reduction in the volume is also associated with a decrease in memory performance in humans and animal models (Driscoll et al., 2006; Hardcastle et al., 2020; O’Shea et al., 2016; Persson et al., 2012; Schuff et al., 1999).

Studies in humans, non-human primates, and rodents have shown that the thinning of the EC and HC during normal aging is not related to neuronal loss; instead, it may be associated with neuronal size or a reduced complexity of dendritic and axonal interactions in the neuropil (Freeman et al., 2008; Merrill et al., 2000, 2001). Within the EC, neurons in layer II are particularly susceptible to harmful consequences of aging, mild cognitive impairment, and AD (Stranahan and Mattson, 2010). Interestingly, in postmortem tissue from individuals ≥80 years old with exceptional episodic memory, called SuperAgers, the soma size of EC neurons (stellate cells) from layer II is larger compared to individuals 20–30 years younger (Nassif et al., 2022). The selective vulnerability of the EC cell population to neurofibrillary tangle (NFT) formation during normal aging and early stages of AD (Braak and Braak, 1985, 1991) was not observed in SuperAgers individuals. NFT burden in SuperAgers was unusually low in EC, with approximately three times fewer NFTs compared with normal elderly across the entire EC (Gefen et al., 2021). Thus, the integrity of EC layer II may be a biological substrate of exceptional memory in old age.

Interestingly, early alterations throughout aging have been observed first in LEC. For instance, reelin, a glycoprotein implicated in synaptic plasticity and expressed by EC layer II neurons, is reduced in an age-dependent manner in LEC but not in MEC, which correlates with cognitive decline in rodents and monkeys (Long et al., 2020; Stranahan et al., 2011). Moreover, long-term potentiation (LTP) induced in the LEC-DG synapses is also impaired in 8-to 12-month-old rats and mice, an alteration that is accompanied by the reduction of TrkB receptor levels and the increment of synaptophysin levels (Froc et al., 2003; Amani et al., 2021). This selective alteration in EC is also observed in human subjects. Postmortem studies show tau accumulation in LEC before being observed in MEC (Braak and Braak, 1985). Furthermore, in patients with amnestic mild cognitive impairment, there is a reduction in the volume and activation of LEC, but not in MEC, which correlates with impaired performance on a memory task (Tran et al., 2022). A detailed evaluation of the anterolateral EC (alEC; LEC in rodents) and posteromedial EC (pmEC; MEC in rodents) in older adults, using high-resolution imaging, showed hypoactivity of alEC, but not pmEC, which was associated with behavioral deficits on an object pattern separation task (Reagh et al., 2018). Altogether, this suggests that LEC, rather than MEC, undergoes early structural and functional changes associated with impaired memory function during the aging process. Therefore, early evaluation of LEC function may help to identify early signs of cognitive decline during aging.

In the HC, cellular, molecular, structural, and functional age-dependent alterations are also observed, however, some of them seem to be unique to specific hippocampal regions. For instance, synaptic loss in the HC highly correlates with cognitive decline during aging and AD (Burke and Barnes, 2010; Colom-Cadena et al., 2020), nevertheless, it seems to be region-specific. Specifically, age-related synaptic loss is observed in EC-GD synapses in rats (Geinisman et al., 1992) and in older adults with memory function decline (Yassa et al., 2010, 2011b). Similarly, synaptic loss is observed in EC-CA3 synapses but not in EC-CA1 or Schaffer collateral-CA1 synapses (Smith et al., 2000; Geinisman et al., 2004; Buss et al., 2021), suggesting a lower age-dependent susceptibility to synapses loss in CA1 area.

In DG, in addition to synaptic loss, adult neurogenesis may be an additional factor underlying cognitive decline during aging. Studies in mammals, including non-human primates, and humans show an age-dependent decrease in adult neurogenesis (Kuhn et al., 1996; Aizawa et al., 2009; Amrein et al., 2011; Spalding et al., 2013; Boldrini et al., 2018; Moreno-Jiménez et al., 2021), which correlates with cognitive decline in rodents and non-human primates (Aizawa et al., 2009). Whether this occurs in humans, is still under debate, we need methodological approaches that allow us to evaluate this process across the lifespan.

The processing of memory formation likely requires coordinated patterns of neuronal activity among brain regions. Balanced excitation and inhibition are crucial for adjusting neural input/output relationships between regions. However, this balance is altered by aging in DG/CA3 regions. Specifically, there is an DG/CA3 increased activity associated with age-dependent memory loss in humans and rodents (Wilson et al., 2005; Patrylo et al., 2007; Miller et al., 2008; Yassa et al., 2011a). This hyperexcitability is likely the result of reduced inhibitory input into the CA3 auto-associative recurrent collateral network and the reduction from DG (Wilson et al., 2005; Buss et al., 2021), which coupled with the synaptic loss of EC-CA3 synapses (Smith et al., 2000) may underly the mechanism for the learning deficit observed during aging.

Meanwhile, in CA1, which resists synapse loss, mechanisms such as Ca^2+^ signaling may also contribute to age-related cognitive impairment. Specifically, it has been shown that aged CA1 pyramidal cells have higher Ca^2+^ conductivity due to the higher density of L-type Ca^2+^ channels, which may lead to disrupted Ca^2+^ homeostasis, contributing to age-associated synaptic plasticity and cognitive deficits (Thibault and Landfield, 1996; Foster and Norris, 1997; Veng et al., 2003; Pereda et al., 2019).

Thus, the variety of alterations in the EC-HC circuitry, some of them region-specific, are likely to contribute to the reduction in hippocampal synaptic plasticity leading to age-related cognitive decline. The participation of neuromodulators in the alteration of the EC-HC circuit during aging, mainly in regions with more vulnerability such as LEC, CA3, and DG, is largely unknown. Therefore, studies analyzing the participation of cortical and subcortical brain regions modulating the EC-HC are needed for a better understanding of the underlying mechanism of age-related cognitive decline.

In the HC, synaptic loss highly correlates with cognitive decline during AD (Colom-Cadena et al., 2020). This cognitive decline is thought to be due to the loss of afferents from EC layer II neurons that span to the outer molecular layer of the DG (Scheff et al., 2006). In neurodegeneration, such as AD, there is a significant neuronal dysfunction with neuronal death and pathophysiological changes (Choonara et al., 2009; Lamptey et al., 2022). In AD, the entorhinal–hippocampal circuit is one of the first circuits impaired in preclinical stages of the disease before cognitive impairments are visible (Igarashi et al., 2014; Nakazono et al., 2018; Jun et al., 2020; Hernández-Frausto et al., 2023; Igarashi, 2023). More than 60% of patients with AD show impaired spatial memory formation and recall. Interestingly, the entorhinal–hippocampal circuit is highly susceptible to accumulation of amyloid-*β* aggregates, NTFs, intracellular tau, and axonal degeneration from early periods (Hope et al., 1994). Moreover, consistent with different mouse models of AD, there are early signs of neurodegeneration observed as an impairment of grid cell formation in MEC. This early sign correlates with a spatial memory impairment linked to hippocampal function (Ahnaou et al., 2017; Igarashi, 2023; Jun et al., 2020; Klein et al., 2016; Mackenzie-Gray Scott et al., 2022; Price et al., 2001; Scheff et al., 2007; Takahashi et al., 2010; Ulm et al., 2021).

In line with this observation, not only the entorhinal cortico-hippocampal circuitry is highly susceptible to amyloid deposition and tau aggregates. Evidence shows that cholinergic, noradrenergic, and dopaminergic fiber nubs also present signs of degeneration in the early stages of AD. Indeed, ACh dysfunction is one of the first theories linked to AD because MS and cholinergic neurons are particularly vulnerable to tau aggregates and neurodegeneration, both of them influencing the entorhinal–hippocampal processing as we discussed previously (Chen Z. R. et al., 2022; Igarashi, 2023; Salimi-Nezhad et al., 2023). Similar to the entorhinal–hippocampal circuit, the LC area has been gaining interest among the scientific community due to the high susceptibility to neurodegeneration in the early periods of AD. The tau accumulation in the LC and the noradrenergic input loss into the EC-HC circuitry may lead to the early signs of tauopathy observed in AD (Chen Y. et al., 2022; Igarashi, 2023). In recent years, the dopaminergic system has taken an increased interest as a promising target to alleviate cognitive deficits during AD course. An early and progressive dysfunction of VTA has been observed in AD. Furthermore, it has been observed that the neuronal degeneration of VTA induces hippocampal hyperexcitability in a mouse model of AD. However, due to its novel finding in AD progression, future studies are needed to unmask the contribution to the EC-HC connectivity function (Igarashi, 2023; Nobili et al., 2017; Shaikh et al., 2023; Spoleti et al., 2024) (Figure 2).

**Figure 3:**
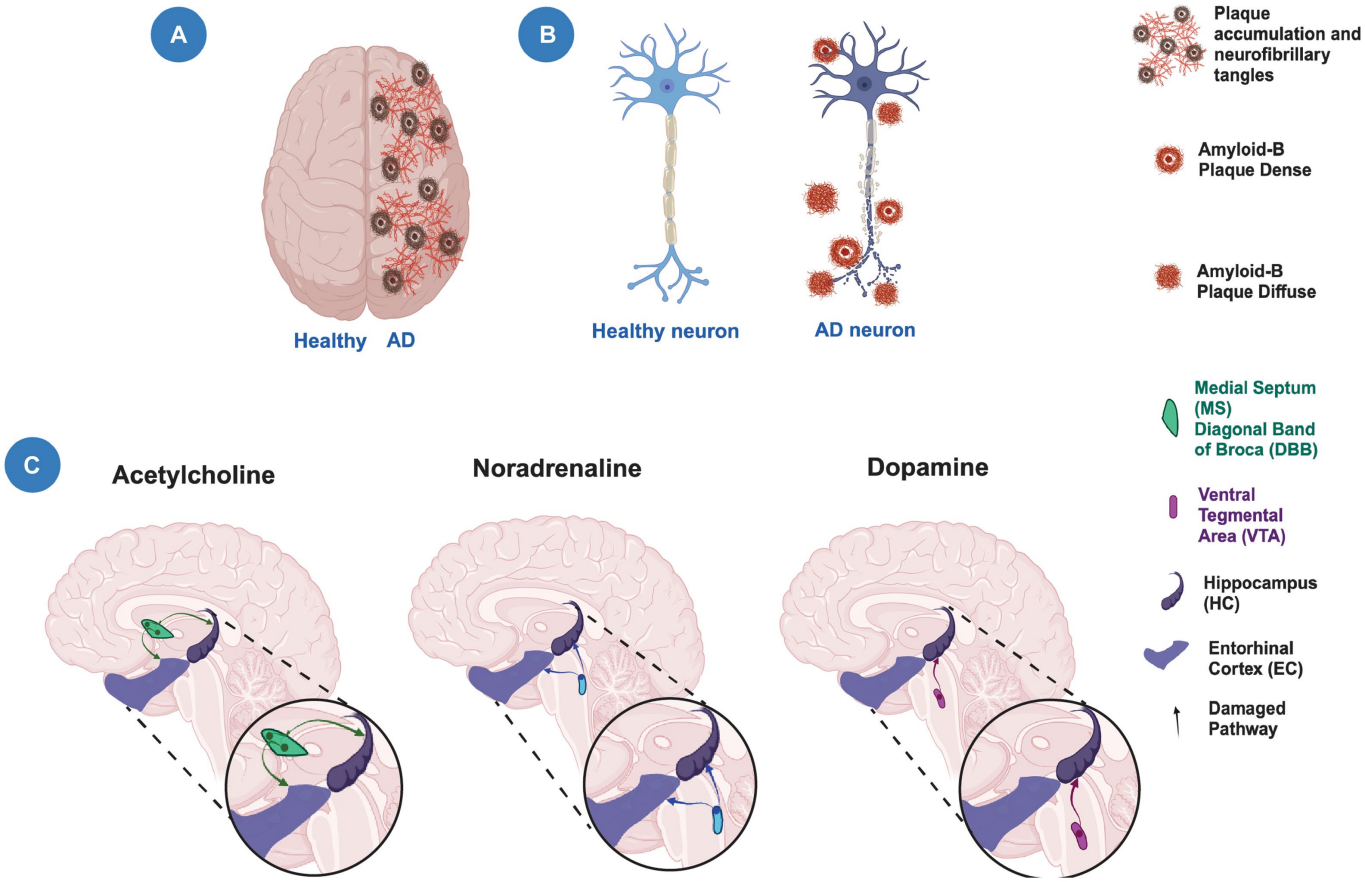
Neuronal degeneration and neuromodulation alterations in Alzheimer’s disease. **(A)** Schematic representation of a human brain showing a healthy hemisphere (left) and a hemisphere in Alzheimer’s disease (AD) pathology (right). The right hemisphere shows the presence of plaque accumulation and neurofibrillary tangles. **(B)** Schematic representation of a healthy neuron (left) and a neuron with neurodegeneration (right) showing the presence of amyloid plaques and axonal damage. **(C)** The modulatory cholinergic and noradrenergic inputs from the Medial Septum/Diagonal Band of Broca (MS/DBB) and Locus Ceruleus (LC), respectively, to the entorhinal cortex-hippocampal circuit are damaged (faded lines) during the temporal course of AD. The modulatory dopaminergic input from the Ventral Tegmental Area (VTA) to the hippocampus is also altered (faded lines). Created with BioRender.

Altogether, this suggests that structural, cellular, and molecular alterations of the EC and HC lead to modifications in the circuitry that contribute to a decline in episodic memory during aging and AD (Figure 3). Moreover, alterations in cortical and subcortical brain regions that modulate the EC-HC circuit may also contribute to cognitive decline. Further studies are needed to determine the contribution of these alterations to the decline in episodic memory during aging and AD and thus prevent, as in SuperAgers, or delay cognitive decline.

## 6 Physical exercise to prevent the entorhinal–hippocampal circuit neurodegeneration

By preventing or delaying cognitive decline, we may be able to restrict the time with dementia or AD and perhaps escape from it. Evidence from systematic reviews and meta-analyses supports the beneficial effects of physical exercise (PE) on cognitive functions in healthy young adults and old subjects with or without cognitive impairment (Blondell et al., 2014; Kelly et al., 2014; Ludyga et al., 2016; Gomes-Osman et al., 2018). While some studies fail to demonstrate PE effects on cognition (Barnes et al., 2013; Sink et al., 2015). The variability in the exercise cognitive effects may be influenced by the differences in duration, exercise training regimen, fitness level, and precise cognitive outcome measures, among other parameters. Despite these differences, it has been shown that regular PE provides a wide range of health benefits affecting nearly all organ systems, inducing the improvement of health or reducing the risk of disease (Booth et al., 2012; MoTrPAC Study Group, Lead Analysts, and MoTrPAC Study Group, 2024).

Physical exercise elicits functional and structural changes throughout the brain. Among specific brain regions, the HC seems to be the key region responsive to PE (Barha et al., 2020; Firth et al., 2018), probably because of its plasticity and susceptibility to age-related atrophy (Fjell et al., 2014) or because most of the research has focused on it due to the HC role in memory functions. However, brain areas such as the EC (Glutamate/GABA), MS/DBB (ACh/GABA), VTA (DA), and LC (NA/DA), which innervate and modulate the HC processing, also experience PE-induced modifications (Ahmad et al., 2009; Segal et al., 2012; Hall and Savage, 2016; Whiteman et al., 2016; Medrano et al., 2021) that may jointly contribute to mediate the positive effects of PE in memory functions. For example, long-term PE increases muscarinic receptor density and high-affinity choline uptake in the HC and the number of cells expressing choline acetyltransferase (ChAT) in the horizontal DBB (Ang et al., 2006; Fordyce and Farrar, 1991), which may modulate the integration of the information in the entorhinal cortico-hippocampal circuit to improve memory functions. Moreover, it has been shown a positive correlation between cardio-respiratory fitness and the volume of the EC and memory performance (Whiteman et al., 2016). However, if we take into account the PE-induced modifications to the first-or second-order presynaptic areas innervating the HC, such as the perirhinal cortex that innervates LEC or prefrontal cortex that innervates LC and VTA (Hopkins and Bucci, 2010; Byun et al., 2024), we may realize PE may indeed induce beneficial effects to nearly the whole brain. How the second-or third-order presynaptic areas to HC modulate the information processing under exercise conditions is still unknown. This information may help to understand the circuits involved in age-dependent decline in memory function and identify very early signs of cognitive deterioration during the aging process, which also contribute to identifying possible therapeutic strategies to improve memory function.

So far, studies in rodents show that in the HC, PE induces molecular, cellular, structural, and functional modifications that are mediated by changes in neurotransmitter levels, blood flow, upregulation of growth factors such as brain-derived nerve growth factor (BDNF), and the increment of dentate adult-born neurons (see, for review, Duzel et al., 2016; Voss et al., 2013, 2019; Vivar et al., 2013). Moreover, PE increases dendritic complexity and the number of dendritic spines in the DG (Eadie et al., 2005), CA1, and EC (Stranahan et al., 2007). Meanwhile, in humans, chronic PE increases cerebral blood volume, hippocampal volume, and perfusion, modifications that are associated with greater BDNF serum levels and improved memory functions (Burdette et al., 2010; Erickson et al., 2011; Pereira et al., 2007; Voss et al., 2019). Additionally, acute PE or even a short bout of mild PE improves DG-mediated pattern separation, memory flexibility, and an increase in functional connectivity between hippocampal DG/CA3 and cortical regions in humans (Suwabe et al., 2017a,b, 2018).

Dentate adult neurogenesis is highly responsive to environmental and physiological factors. Particularly, PE increases cell proliferation and adult neurogenesis and increases memory performance in rodents (van Praag et al., 1999; Uda et al., 2006). Furthermore, 1 month of PE reorganizes the circuitry in which adult-born granule cells are integrated (Vivar et al., 2016). It includes the increment of connectivity from EC (LEC and caudomedial EC), MS, and supramammilary nucleus (Vivar et al., 2016). Moreover, when PE is extended to more than 6 months, the circuitry of “old” adult-born granule cells (6–9 months old) born during early adulthood showed additional modifications. Specifically, PE increased the inputs from hippocampal INs to “old” adult-born granule cells (Vivar et al., 2023), which may reduce aging-related hippocampal hyperexcitability (Wilson et al., 2005). PE also prevented the loss of adult-born granule cell innervation from the perirhinal cortex and increased the input from the subiculum and EC (Vivar et al., 2023), brain areas that are essential for contextual and spatial memory. Therefore, long-term PE could maintain the wiring of adult-born granule cells born during early adulthood, which may function as a cognitive reserve during the aging process. Whether the circuit of granule cells born during development is modified by PE is still unknown. However, this evidence supports the idea that PE may also induce modifications to brain areas innervating the HC, which may contribute to memory function improvement and preservation during aging.

## 7 Conclusion

Recent evidence supports the idea that the EC in the LEC and/or MEC portion and the HC area receive robust inputs from neuromodulators such as acetylcholine, noradrenaline, and dopamine brain centers playing an important function in memory processing. It remains unclear how these neuromodulator centers can indirectly modulate the entorhinal cortico-hippocampal circuit through the canonical-glutamatergic or non-canonical GABAergic pathways in non-pathological conditions. Furthermore, due to the importance of the entorhinal cortico-hippocampal circuit in aging and AD pathogenesis, it is important to understand the underlying mechanisms of memory processes and its modulation in early periods of aging and before the onset of the AD pathogenesis as a possible target to stop or prevent the progression of the cognitive impairments. Finally, PE may be used as a tool to prevent and/or delay cognitive decline. The growing number of studies in human and animal models support the beneficial effects of PE on memory processing. Physical exercise induces a marked reduction of risk of neurodegeneration and prevents functional and anatomical deterioration. A link between neuromodulation and PE in the entorhinal cortico-hippocampal circuit, as a possible instrument to prevent and decrease neurodegeneration, remains unidentified.
